# Review of Two Decades of Cholera Diagnostics – How Far Have We Really Come?

**DOI:** 10.1371/journal.pntd.0001845

**Published:** 2012-10-11

**Authors:** Michal H. Dick, Martine Guillerm, Francis Moussy, Claire-Lise Chaignat

**Affiliations:** 1 Medical School, Australian National University, Canberra, Australia; 2 United Nations International Children's Emergency Fund/United Nations Development Program/World Bank/World Health Organisation Special Programme for Research and Training in Tropical Diseases, World Health Organization, Geneva, Switzerland; 3 Global Task Force on Cholera Control, Department of Public Health and Environment, Health Security and Environment, World Health Organization, Geneva, Switzerland; Massachusetts General Hospital, United States of America

## Abstract

**Background:**

Cholera, an ancient scourge, continues to inflict high rates of mortality today. The rising incidence of epidemics in areas of poor sanitation and crowding highlight the need for better epidemic prevention and early response. Such interventions require the availability of rapid and accurate diagnostic techniques to trigger timely response and mitigate the scale of the outbreak. The current gold standard of bacterial culture is inadequate for rapid diagnosis, highlighting the overarching neglect of field diagnostic needs. This paper was written to support the World Health Organisation's Global Task Force on Cholera Control mandated Cholera and diarrhoeal disease laboratory Network (CholdiNet) in devising a protocol for the validation of Rapid Diagnostic Tests (RDTs) for *Vibrio cholerae*. The status of diagnostic tools for *Vibrio cholerae* is assessed, describing products that have been commercialised over the last two decades and discussing their peer-reviewed evaluation.

**Method:**

Review of post-1990 peer-reviewed and grey literature on rapid diagnostic tests for *Vibrio cholerae*.

**Results:**

Since 1990, twenty four diagnostic tests have been developed for the detection of *Vibrio cholerae* in human faecal samples. Fourteen of these have also been described in the literature, with rapid chromatographic-immuno assays (CIA) featuring strongly. Polymerase chain reaction (PCR) assays maintain the ability to detect the lowest amount of bacteria; however CIAs achieve both low detection thresholds and high sensitivity and specificity, making them possible candidates for use in field conditions. Field and laboratory studies were performed in a wide range of settings demonstrating variability in performance, however only a few of these studies were sufficiently stringent, highlighting five RDTs that showed promise in field conditions; COAT, IP cholera dipstick, SMART, IP dipstick and Medicos. In light of non-independent reporting, the authors would like to see these five products undergoing additional studies, with further technical improvements if needed and commercial production. The authors hope that public health use of such a RDT in limited-resource field conditions on stool samples may contribute to effective reduction in cholera epidemic spread.

## Author Summary

Rising prevalence of cholera outbreaks highlights the need for accurate detection tools. Diagnosing cholera early at the onset of an epidemic, at field level, should allow for a more timely response and a quick containment of the spread and thus a diminished case load. Currently the gold standard to identify the bacteria, Vibrio cholerae, from patient samples remains reliant on lengthy bacterial cultures and an array of biochemical tests. Furthermore, the need for highly-skilled operators and numerous laboratory resources underline the inadequacy of sophisticated tests for use in remote locations. Research to develop more appropriate tools has largely focused on rapid diagnostic tests and attempts to simplify existing technologies. This is yet to deliver evidence-based appropriate tools to address the burden-of-disease cholera inflicts. In light of this neglect we have taken the first step, assessing developments in commercialised diagnostic tools, reviewing previous evaluations undertaken in the literature since 1990. In doing so, we highlight evaluation study parameters that could benefit from stringent standardisation, and identify five tests that show promise for use in field conditions. The authors recommend an indipendent assessment of these products, including technical improvements as required and production to trigger early detection of cholera epidemics.

## Introduction

Cholera is an infectious disease caused by the bacteria *Vibrio cholerae* O1 and/or O139. When ingested, its clinical sequelae include the acute onset of severe secretory ‘rice water’ diarrhoea. Within three to four hours of symptom onset, a previously healthy individual may become severely dehydrated and if not treated may die within twenty four hours. This makes cholera one of the most rapidly fatal infectious illnesses known whose clinical management, simple rehydration, can be instituted empirically and remains cheap, safe and life-saving [1].

Cholera inflicts a heavy economic burden in its endemic settings due to its rapid spread and ability to cause large epidemics [2]–[3]. Since the first reported epidemic in the 19^th^ century on the Indian subcontinent, cholera has spread to all inhabited continents and has become endemic in Africa, South and East Asia [4]–[5]. In recent years, reported cholera cases increased steadily reaching more than 300' 000 cases including more than 7'500 deaths during 2010 [6]. During the same year and for the first time since 1995, the proportion of global cases reported to WHO from the African continent declined from more than 90% to less than 50%, a consequence of the large cholera outbreak which occurred in Hispaniola.

Recent trends suggest that the number of outbreaks of cholera will continue to increase in vulnerable areas in the future. As populations of poor countries continue to coalesce in mega-cities with poor sanitation and people move rapidly around the globe, new and more virulent strains of *V. cholerae* are expected to disseminate more rapidly [7]–[8]. The unpredictable emergence and spread of antibiotic-resistant strains, together with increasing severe weather events and changes in water temperature and nutrient levels means that the occurrence of more frequent cholera outbreaks may continue to occur in the foreseeable future [9].

To combat this threat, attention in clinical and public health circles has expanded to encompass efforts to improve sanitation and utilization of new vaccines to improving epidemic response through early detection. By detecting cholera outbreaks as early in their course as possible additional resources can be more rapidly brought to bear to mitigate the size, scope and duration of the outbreak and subsequent spread of the illness. In addition, using a cheap rapid diagnostic test on watery stool samples, under field conditions with limited resources holds great potential to aid cholera control efforts, not because empirical treatment or later treatment is less effective but rather because decreasing the numbers of cholera infections and aiding in epidemic-preventing surveillance will free up resources that should be directed towards the excessively complex task of fixing the underlying socio-economic and environmental factors that propagate the spread of cholera. This shifts the focus from response measures, which often arrive too late to halt the course of an epidemic.

With this approach in mind, the current gold standard for laboratory diagnosis of cholera becomes evidently inadequate due to lengthy culturing on selective growth media. Preliminary identification based on colony appearance on Thiosulfate Citrate Bile Salts Sucrose Agar (TCBS) is traditionally confirmed using an array of biochemical tests, taking a few days to confirm a case of cholera and requiring numerous laboratory resources [10]. Such tests still have a role in antibiotic sensitivity surveillance during epidemics, however timely field diagnosis calls for Rapid Diagnostic Tests (RDTs). Most RDTs work by capturing a characteristic component of the cholera bacteria on a solid surface and binding it with specific reagents to produce a visual change, allowing for rapid detection of a cholera infection. Following the principle of commercialised home-pregnancy detection kits, such cassettes and dipsticks (characterised by quick turnover time, ease of use and accuracy) are extending their reach outside classical laboratory networks thereby aiding diagnosis in under resourced field settings. The last twenty years have also seen attempts to modify technologies such as Polymerase Chain Reaction (PCR), Enzyme Linked Immunosorbent Assay (ELISA) and agglutination methods to make them more applicable as triggers for the timely response against cholera.

At the time of writing there is no consensus as to the most efficacious cholera diagnostic for early case detection. Very few diagnostic tests have been well described and throroughly evaluated [11]. This study aims to identify and describe all the commercialised diagnostic tools developed and evaluated since 1990. It then explores the evidence base for these tests, including their sensitivity, specificity and reliability. In doing so the paper exposes the discrepancies that exist between research and field application of these diagnostic products. The authors hope that this work will facilitate future consensus regarding the best diagnostic for early cholera epidemic detection, and hence begin to address the neglect of cholera.

## Methods

**Figure 1:**
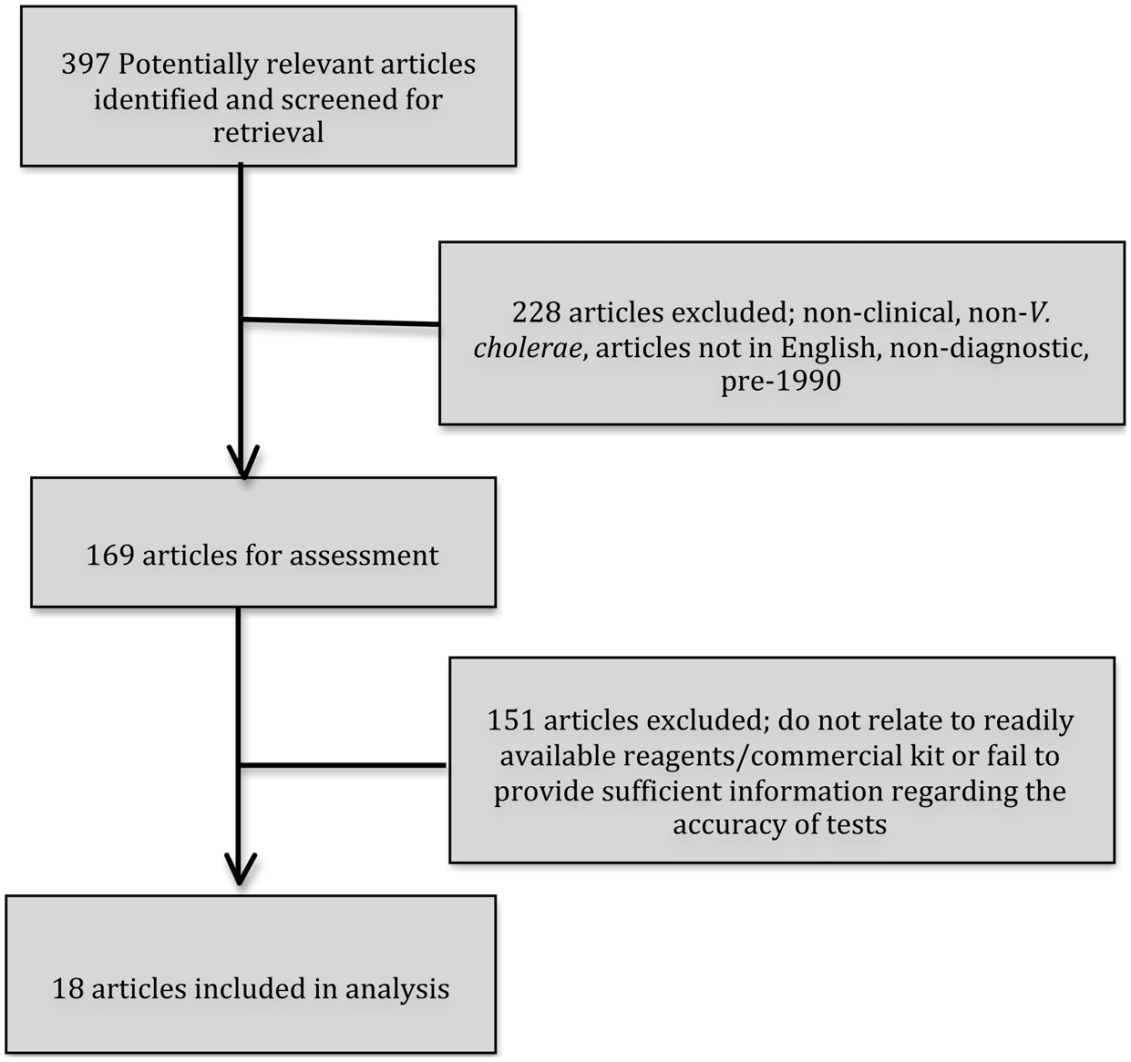
Process of article selection for diagnostic evaluation.

Systematic searches were conducted using PubMed, SCOPUS, EMBASE, LILACS, ScienceDirect, GoogleScholar, Medline Plus, and ResearchGATE. The following search strategy was used: All published English literature since 1990 focusing on diagnostic tools for human clinical samples of cholera, and the evaluation of diagnostic tests, using the initial terms “Cholera”[Mesh] OR “*Vibrio cholerae*”[Mesh] OR cholerae OR Choleras OR cholera OR “Cholera Toxin”[Mesh] AND “Sensitivity and Specificity”[Mesh] OR “Diagnosis”[Mesh] OR “diagnosis”[Subheading] OR (routine AND (test OR tests or testing)) [TIAB] OR (false AND ((positive or positivity) or negative)) [TIAB] OR diagnos* [TIAB] in PubMed. The reference lists of relevant papers were followed and a manual search was conducted of journal titles with multiple publications on the topic, including; Journal of Clinical Microbiology, Transactions of the Royal Society of Tropical Medicine and Hygiene, Biosensors and Bioelectronics, Journal of Microbiological Methods. To elicit information regarding commercialised diagnostic tools, a grey literature search was conducted of manufacturers and governmental regulatory websites, following up manufacturing and product names referred to in the aforementioned literature. The choice to limit the articles to the year 1990 reflects the upsurge of technologies following the South American outbreak of the early 1990s and the discovery of the new strain O139 Bengal in 1992. Furthermore, papers were excluded if they did not focus on diagnosis of human samples of *Vibrio cholerae* (Figure 1).

At the analysis stage, the diagnostic technologies were described in terms of: their detection limit, their diagnostic target, the method used (microscopy, agglutination, ELISA, immunochromatography or PCR), turn around time, intended use and settings of use. The sensitivity and specificity of the tests were reported from laboratory evaluation, highlighting field studies (when performed). These field studies were then filtered for having a sample size greater than 100 stool samples and reporting Positive Predictive Value (PPV) and Negative Predictive Value (NPV) and presented in tabulated form according to the aformentioned values. Following this, a review of the evidence on the validity and reproducibility underpinning the rapid diagnostic tests (RDTs) was performed.

## Results

### Diagnostic Tests

In January 2010, twenty four commercialised diagnostic tests were identified (Table S1). Ten more received a mention in the literature but due to suspected discontinuation of production or fall-back did not provide sufficient information (see note under table S1 for details). Of the twenty four described in detail below, a clear evolution is portrayed following closely the historical development of diagnostic technologies in this field from cell culture and microscopy methods towards agglutination methods, immunochromatographic assays, and Polymerase Chain Reaction (PCR)-based assays. The majority of tests were found to be based on antigen or antibody detection, with a large proportion of the remaining tests being DNA-based. It is important to note that this investigation came across many diagnostic methods that have never been commercialised including those that are currently in the scientific pipeline – technologies such as microarrays and electro-chemiluminescence are being developed as biosensors for cholera toxin and other antigens. Loop-mediated isothermal amplification (LAMP), ELISA and simplified PCR protocols are but a few of the new approaches to the diagnosis of cholera that are likely to be seen in the future, unfortunately these are beyond the scope of this paper.

Table S1 summarises the results in two sections, separating DNA from antigen detection. All of the tools are described according to the information provided by the manufacturer; product parameters, intended application and performance. The details provided should allow the reader to compare both the analytical sensitivity, according to the limit of detection, and test utility according to the turnaround time.

### Diagnostic Evaluations of rapid tests

Since 1990 there have been eighteen laboratory and field evaluations of commercial diagnostic tools for the detection of *Vibrio cholerae* in clinical samples (see Tables S2 & S3) [12]–[29]. These evaluations have been carried out around the world, with a large proportion taking place in the Indian subcontinent. The largest study enrolled around 400 cases and controls, while most other sample cohorts were quite small, the smallest being just 30 patient samples [30]. Tables S2 and S3 summarise the peer reviewed evaluations of the aforementioned diagnostic tests conducted between 1990 and 2008. Table S2 highlights those tests conducted in field conditions. Fourteen different diagnostic tests were found to have been evaluated and are thus compared here. Tables 1 and 2 rank those tests that were evaluated under field conditions with over 100 samples, excluding tests where negative and positive predictive values were insufficiently reported. Overall, there appear to be five such tests; coagglutination test (COAT), Institute Pasteur (IP) cholera dipstick, Sensitive Membrane Antigen Rapid Test (SMART), IP dipstick and Medicos. Tables 3 and 4 similarily present the sensitivities and specificities of the aformentioned eighteen evaluations by rank of test.

**Table 1:** Positive Predictive Value ranking.

Rank	Product name	PPV
1^[18] (Arya)^	COAT	100
2^[15] (Nato)^	IP cholera dipstick	95.6
3^[15] (Nato)^	IP cholera dipstick	94.8
4^[15] (Nato)^	IP cholera dipstick	86
5^[14] (Kalluri)^	SMART	84
6^[14] (Kalluri)^	IP dipstick	83
7^[14] (Kalluri)^	Medicos	71

**Table 2:** Negative Predictive Value ranking.

Rank	Product name	NPV
1^[15] (Nato)^	IP cholera dipstick	100
2^[15] (Nato)^	IP cholera dipstick	98.6
3^[18] (Arya)^	COAT	95
4^[15] (Nato)^	IP cholera dipstick	93.3
5^[14] (Kalluri)^	Medicos	90
6^[14] (kalluri)^	IP dipstick	88
7^[14] (kalluri)^	SMART	84

Tables 1 and 2 highlight those tests that were evaluated under field conditions with over 100 samples, excluding tests where this information was insufficiently reported.DFA = Direct Fluorescent Antibody, IP = Institute Pasteur, COAT = Coagglutination Test, VC = *Vibrio cholerae*, SMART = Sensitive Membrane Antigen Rapid Test.

**Table 3:** Specificity ranking.

Rank	Product name	Value
1^[21] Colwell^	Cholera Screen	100
2^[20] Hasan^	Cholera SMART	100
3^[16] Hasan^	Bengal Screen	100
4^[17] Islam^	Cholera SMART	100
5^[19] Hasan^	DFA COLTA	100
6^[18] Arya^	COAT	100
7^[20] Hasan^	Cholera SMART	100
8^[15] Nato^	IP cholera dipstick	96
9^[14] Kalluri^	SMART	95
10^[15] Nato^	IP cholera dipstick	92.5
11^[16] Hassan^	Bengal DFA	89
12^[13] Wang^	IP O1 cholera dipstick	89
13^[15] Nato^	IP cholera dipstick	84
14^[14] Kalluri^	Medicos	79
15^[21] Colwell^	Cholera Screen	77.8
16^[12] Harris^	Crystal VC	71–76
17^[14] Kalluri^	IP dipstick	67
18^[21] Colwell^	Cholera Screen	60

**Table 4:** Sensitivity ranking.

Rank	Product name	Value
1^[15] Nato^	IP cholera dipstick	100
2^[16] Hassan^	Bengal DFA	100
3^[21] Colwell^	Cholera Screen	100
4^[20] Hasan^	Cholera SMART	100
5^[19] Hasan^	DFA COLTA	100
6^[17] Islam^	Cholera SMART	100
7^[15] Nato^	IP cholera dipstick	98.5
8^[21] Colwell^	Cholera Screen	98
9^[12] Harris^	Crystal VC	97
10^[20] Hasan^	Cholera SMART	95.6
11^[13] Wang^	IP O1 cholera dipstick	95
12^[16] Hasan^	Bengal Screen	95
13^[15] Nato^	IP cholera dipstick	94.2
14^[18] Arya^	COAT	92
15^[21] Colwell^	Cholera Screen	85.7
16^[14] Kalluri^	Medicos	84
17^[14] Kalluri^	IP dipstick	83
18^[14] Kalluri^	SMART	58

Tables 3 and 4 list cholera diagnostic tests' specificity and sensitivity values as reported by field and laboratory evaluations. SMART = Sensitive Membrane Antigen Rapid Test, DFA = Direct Fluorescent Antibody, IP = Institute Pasteur, COAT = Coagglutination Test, VC = *Vibrio cholerae*.

## Discussion

Alongside other diarrheal diseases, cholera is a major cause of preventable childhood and adult deaths in developing countries [31]–[32]. Given international attempts like the fourth millennium development goal (MDG) to reduce such burdens, it is surprising that cholera has not gained more public health attention. Availability of rapid diagnostic tests would be extremely useful for diagnosing cholera epidemics early during their onset in the field, using watery stool samples from patients at the bed side. Yet out of 15.7 million US dollars spent by US government in 2007 on cholera research, only 1% went to diagnostic testing. The scale of scientific spending on the development and implementation of diagnostic and therapeutic intervention for cholera is disproportional to the impact of the disease [33], moving the authors to conclude that cholera is a ‘neglected disease’. [34].

On the background of such neglect, the authors found twenty four commercialised diagnostic tools for the detection of *Vibrio cholerae* in patient samples. The majority of these tests were of the rapid detection kind, utilising immunochromatography, direct fluorescence or agglutination to identify *Vibrio cholerae* antigens in stool samples. Due to the reality of limited resource availability, it would be logical to choose the best tests from those already available, based on pre-existing evaluations. At the time of writing, the authors could not find any literature reviews of diagnostic tools comparable to the scale of this study. At best, papers describe the comparison in efficacy between three diagnostic tools [14]. Those aforementioned diagnostic evaluations appear to have a number of limitations. An overarching problem is the lack of standardisation of assessment criteria, making comparison between different diagnostic tests very difficult. This issue has been addressed previously in the Diagnostic Evaluation Expert Panel (DEEP) guidelines which provide guidance towards an improved assessment of the performances of many diagnostic tests in the case of malaria, dengue, visceral leishmaniasis and tuberculosis. [35]. A number of critical parameters should be carefully assessed when designing a diagnostic test evaluation (summarised in table 5).

**Table 5:** Recommendations made to address shortcomings of diagnostic test evaluations.

	PROBLEMS	PROPOSED SOLUTIONS
	STUDY DESIGN	
1	Small sample size• Limits validity and reproducibility	Larger studies are feasible
2	Variable sample type• Fresh vs. archives	Controversial
3	Gold standard• Uncertain standard case definition• Newer tests outperforming old	Recommend reviewing gold standard according to accuracy, cognisant of different uses for different contexts
4	Testing stools vs. purified cultures• Affects specificity	Stool samples should be used
5	Not approximating field conditions• Population descriptors are essential for PPV, NPV	Population descriptors should be reported for every population studied
6	Specificity• Limited data against common gut organisms	Specificity testing against confounding gut microflora should be considered prior to commercialisation
	TEST DESCRIPTORS	
7	Little data available on stability• Importance of climate variations i.e. temperature, humidity on shelf life and accuracy	Manufacturers should perform stability studies (real time preferably)
8	Reporting of reproducibility• Between different users• As an inherent quality of the tools• Assessment of manufacturing and inter-lot variation	User reproducibility reported between at least three users and with sufficient number of samples
	SCIENTIFIC RIGOR	
9	Conflation sensitivity with lowest detectable dilution	Scientific rigor should be a criteria for further inclusion and re-evaluation of test

One implication of this critique of present evaluations is that even though it would appear at first that a particular test has the best PPV, NPV, specificity or sensitivity, the limitations of the evaluations means that no one diagnostic can be recommended without further improvement and standardized evaluation.

### Intended use

It is important to note that the scientific community remains divided regarding the intended use of rapid cholera tests, failing to provide any clear consensus about the requirements for such a rapid diagnostic test. In trying to formulate such requirements one must recognise the stark difference between individual diagnostic utility and early detection of epidemic or other public health roles. This preliminary step is critical as the characteristics of the tests are expected to differ in each scenario. In the case of PCR technologies, they currently have an important role in individual diagnostic confirmation but also play a role in outbreak detection in combination with RDTs through epidemiology and surveillance. PCR methods have shown to be advantageous at being particularly sensitive to small amounts of infectious bacteria and having the added value of characterising the strains of the epidemic during the same diagnostic investigation. The tests in table S1 detect a range of genes including those that differentiate between biotype El tor and classical cholera, the serotype specific *wbe* and *hemA* genes and virulence genes for cholera toxin (*ctx*), zonula occludens toxin (*zot*), accessory cholera enterotoxin (*ace*) and a tetracycline resistant genotype (*tetA*). Such tests could be of great utility if they continue in their adaptation for field use, especially in surveillance programs within the reach of reference laboratories. Their limited value in field settings highlights that a single test cannot serve all contexts and that the end user should be clearly informed about the limitations of each technology.

### Study design

As stipulated in the DEEP guidelines, the study design is the first parameter to consider to ensure that an evaluation study will answer the intended question, be it a lab evaluation or field testing. NPV and PPV can only be derived from field testing when disease prevalence is known. It is therefore critical that studies analysing cholera diagnostic tests mimic the conditions the tests are designed to be used in. Among the rapid diagnostic test evaluations, only a limited number of studies were conducted in field conditions on fresh stool samples with provision of population descriptors. Such demographics may ensure that tests are really applicable to those who suffer from cholera. Therefore it is not unreasonable to suggest that current tests may encounter unexpected results when taken to the field. In the studies analyzed, recurrent conflation of the term ‘sensitivity’ to describe the lowest detectable dilution in the stools made it more difficult to carry out an accurate comparison of the tests. However, overall, some of the new rapid tests (Smart Q – 10^5^ CFU/mL) are performing to the same detection standards as the PCR based tests (BAX - 10^4^ CFU/mL).

### Sample size and target population

A key study design parameter is the sample size of the investigation. A small sample size limits the validity of the evaluation and the reproducibility of the results. This was the case in most of the evaluations examined, suggesting a direct impact on their specificity and sensitivity measurements. Indeed many of the studies were found to have 100% sensitivity or specificity using approximately 100 participants. In spite of this caveat, it is important to recognize that due to the challenge of long-term preservation of cholera organisms in stools specimen sample archives are hard to come by and conducting the study on larger sample size of fresh faecal samples in a resource-poor setting is challenging. However, in the broader literature covered during the course of this analysis, four larger cohorts were found, each reviewing tests that have not yet to our knowledge become commercialised (hence being excluded from the final analysis) [36]–[38]. The largest investigation by far was a collection of 6,497 hospital patients by Chaicumpa et al in 1998 [39] suggesting that such numbers are not impossible and that current sub-standard sample sizes should not be allowed to become the acceptable norm.

Another point to consider is whether sample archives are ideal for a disease like cholera, and whether greater benefit may be gained from the acquisition and analysis of fresh samples. For example, if fecal material from children was under-represented in the study population, health workers may find that the tests are not as accurate or more prone to diagnostic errors. Such errors relate to our ability to infer test efficacy from the field data. Since positive predictive values and negative predictive values are dependent on the prevalence of the disease in the population, a factor that can differ between different age groups, the utility of these tests may differ for children. As children tend to experience greater mortality in association with severe cholera, it is paramount to diagnose the onset rapidly.

### Choice of gold standard

Studies lacked a consistent comparison to any one gold standard, many studies neglecting to undertake detection by both test and reference methods across the sample range to confirm the validity of the result. The variability in the use of sample panels and study populations underlined the lack of a standardised case definition. The absence of a clear benchmark for assessing the tests is made worse by the newer tests performing better than the gold standard, leaving no adequate comparator. Furthermore, some of the diagnostic tests were tested against purified cultures of cholera. This fails to assess how they would fare when used with real patient stool samples packed with gut micro flora and likely to have many other contaminating interactions. Therefore, if used, the results of this investigation are only valid for the use of the diagnostic after culturing, hence defeating the purpose of making these into rapid diagnostic tests.

### Test panel composition and challenge panels

Following the same principle, many of the tests appear to be rather specific on first glance, however only few were thoroughly tested against confounding microorganisms commonly found in stool samples which could lead to a similar case presentation of watery stools. Some studies went so far as to not utilise a negative control. Testing performance against a well characterised challenge panels is required.

### Robustness of the product: reproducibility and heat stability

Data showing robustness of the test design are important to support deployment in the areas of highest need. Test descriptors such as a test's reproducibility were markedly underreported; lacking rigorous replication important for assessing the inherent quality of the diagnostic tool, the reliability of different production lots and the ease of replication by different users. This also reflected the lack of important mention of test heat stability. A test's intended use is directly linked to analytical parameters such as heat stability. In the case of cholera much of its endemic settings are affected by harsh temperatures and humidity, factors which must be taken into account for the shelf life and accuracy of the test.

It is important to note that this is not a true systematic analysis, nor did the analysis review the entire literature, the authors selected to review only articles written in English from the year 1990 onwards. In spite of the aforementioned limitations of these studies (see table 5), the authors present here a first attempt to rank the existing tests according to sensitivity and specificity or PPV and NPV (see tables 1–4). In doing so we have identified that only five tests; COAT, IP cholera dipstick, SMART, IP dipstick and Medicos have been evaluated under field conditions, with a large enough sample size, providing data for essential evaluation parameters. In light of these products' low specificity and sensitivity findings (some less than 90%) and non-independent reporting of the data the authors strongly recommend further studies assessing field performance of these promising tests. International collaboration is called for to co-ordinate well designed evaluation studies with standardized protocols, using best gold standard and assess the test performance for the intended use they were built for. From robust data sets, the scientific community can then derive performance benchmarks for each intended use of the available commercial cholera diagnostic tests.

Caveats in the available information make it harder to make overarching comparisons between the different diagnostic tools, and so does the redundancy in product re-packaging under different names. The neglected nature of cholera in terms of the ratio of its burden to the relative attention it gets (funding, awareness, research) means that most of the diagnostic tools developed thus far have never progressed to mass production and can only be found in small batch numbers for a short period of time. This makes any future evaluation of these tools fraught with difficulties. Much of the information about the respective diagnostic tools has been provided by manufacturers in media releases and product information and is therefore often lacking in reliable information regarding the testing protocol. These are aspects that a future evaluation could address by carrying out independent testing of the products, facilitating an evidence-based revision of the diagnostic strategies for cholera. The authors recommend a prioritization of research and development agenda, and would like to see the five promising field evaluated products highlighted in this article (table 1 and 2) undergoing further independent evaluation, followed by technical improvements if needed and production for use in field condition. Ascertaining the best RDT for early detection of epidemics in this way will maximize the benefits using constrained financial resources available.

Despite the limitations of this review, it is the first time a comprehensive picture of the market of diagnostic tests for cholera has been revealed. In doing so, many of the criteria for more thorough investigation have been elucidated and will likely be incorporated into future standard formulations of evaluation guidelines. The authors hope that the availability and use of the resulting reliable and user friendly rapid diagnostic tests will allow for the triggering of timely response to outbreaks and thus limit spread and burden of cholera.

**Box I – Key Points**

- 24 Cholera diagnostics tests have been developed since 1990, 14 of which have been reviewed in 18 peer-reviewed analytical papers.
- The majority of the tests were RDTs and PCR technologies, others include agglutination and direct fluorescence antibodies.
- Overall the quality of peer-reviewed evaluations of diagnostic tests for cholera is problematic with issues raised in regards to sample size, sample types, gold standard, context suitability for the testing of rapid diagnostic tests and insufficient information provision for test descriptors.
- DEEP guidelines propose a roadmap for standardised evaluation studies of diagnostic technologies and ought to be used to further evaluate the five RDTs that have shown promise under field conditions; COAT, IP cholera dipstick, SMART, IP dipstick and Medicos.
- The limited research and funding in this field highlight the neglect of cholera and calls for increased coordination and prioritisation in the domain of R&D for cholera diagnostic tests to promote early epidemic prevention.

**Box II –Key papers**

- Banoo S, Bell D, Bossuyt P, Herring A, Mabey D and Poole F et al (2010). Evaluation of diagnostic tests for infectious diseases: general principles. Nature Reviews Microbiology; 8(12): S17–S29.
- Bhuiyan NA, Qadri F, Faruque AS, Malek MA, Salam MA, et al. (2003). Use of dipsticks for rapid diagnosis of cholera caused by *Vibrio cholerae* O1 and O139 from rectal swabs. Journal of Clinical Microbiology; 41: 3939–3941.
- Harris JR, Cavallaro EC, de Nobrega AA, Dos S Barrado JC, Bopp C, et al. (2009). Field evaluation of Crystal VC Rapid Dipstick test for cholera during a cholera outbreak in Guinea-Bissau. Tropical Medicine & International Health; 14(9): 1117–21.
- Kalluri P, Naheed A, Rahman S, Ansaruzzaman M, Faruque AS, et al. (2006). Evaluation of three rapid diagnostic tests for cholera: does the skill level of the technician matter? Tropical Medicine & International Health; 11: 49–55.
- Nato F, Boutonnier A, Rajerison M, Grosjean P, Dartevelle S, et al. (2003). One-step immunochromatographic dipstick tests for rapid detection of *Vibrio cholerae* O1 and O139 in stool samples. Clinical and Diagnostic Laboratory Immunology; 10: 476–478.

## Supporting Information

Table S1 **Commercial information for marketed diagnostic tools for** ***Vibrio cholerae*** **.**
(DOC)

Table S2 **Recent peer-reviewed evaluations of cholera diagnostic tests - Field.**
(DOC)

Table S3 **Recent peer-reviewed evaluations of cholera diagnostic tests - Laboratory.**
(DOC)
