# Supplementary material for: Review of Two Decades of Cholera Diagnostics – How Far Have We Really Come?
Source: PLoS Negl Trop Dis. 2012 Oct 11;6(10):e1845. doi: 10.1371/journal.pntd.0001845 (PMC3469466; doi:10.1371/journal.pntd.0001845)
Supplement: Table S1 — Commercial information for marketed diagnostic tools for Vibrio cholerae . (DOC) [file pntd.0001845.s001.doc]

Table S1. Commercial information for marketed diagnostic tools for *Vibrio cholerae*

| **Product name** | **Developer** | **Test format** | **Target** | **Intended use** | **Site to be used** | **Limit of detection** | **Turn-Around-Time** |
| --- | --- | --- | --- | --- | --- | --- | --- |
| **Primer Design kit** | Primer Design Ltd | RT-PCR | *ctx*B | In-vitro quantification of *V. cholerae* genome | Reference lab | <100copies target template | <2 hours |
| **Cholera DNA Rapid (^EZ^Cholera Amp)** | MITD, Universiti Sains Malaysia | Thermostable PCR | *lol*B (*hem*A) | Diagnosis in low-cost settings | Peripheral and reference labs | ~50ng for genomic DNA | 3hours |
| **^EZ^Amp Octaplex Cholera** | MITD & INFORMM, USM | PCR | 8 genes (*tet*A, *hem*M, *rfb*, classical O1, El Tor & O139, *ctx, zot, ace*) | Clinical diagnosis and surveillance | Reference and peripheral labs | ~50ng for genomic DNA | 3-4 hours |
| **^EZ^DNA Amp** | MBDr, Malaysia | Thermostable PCR | DNA | Clinical diagnosis and surveillance | Peripheral & reference labs | ~50ng for genomic DNA | 2 hours |
| **Amp Cholera Genosensor** | MBDr, Malaysia | Thermostable PCR | *V. cholerae* O1 (El Tor, Classical, Ogawa, Inaba), O139  and non-O1, non-O139 serogroups. | Clinical diagnosis and surveillance | Peripheral & reference labs | 10 CFU/μL | <8hours (inc 6hr incubation) |
| **Cholera Vibrion O1/0139 Real Time PCR Kit,** | Shanghai ZJ Bio-Tech Co., Ltd., China | RT-PCR | *ctx* gene | Detection *ctx* gene in excreta or water samples | Reference labs | <10^7^copies/mL | 1hr |
| **Cholera Vibrion Real Time PCR Kit** | Shanghai ZJ Bio-Tech Co., Ltd., China | RT PCR | *ctx* gene | Detection *ctx* gene in excreta or water samples | Reference labs | <10^7^copies/mL | 1hr |
| **Cholera Vibrion (Gene CTX) Real Time PCR Kit** | Shanghai ZJ Bio-Tech Co., Ltd., China | RT PCR | *ctx* gene | Detection ctx gene in excreta or water samples | Reference labs | <10^7^copies/mL | 1hr |
| **VET-RPLA toxin detection kit** | Oxoid Inc., UK | RPLA | *V. cholerae* Enterotoxin | Detection in culture filtrates | Peripheral and reference labs | 1-2ng/ml | <24hr |
| **Cholera Screen** | New Horizons Diagnostics Co., USA | COAT | O1 LPS | ** | Field & health posts | ** | 15min |
| **Bengal Screen** | New Horizons Diagnostics Co., USA | COAT | O139 LPS | ** | Field & health posts | ** | 15min |
| **IP dipstick** | Institute Pasteur, France  [not commercialized] | CIA | O1 LPS | Rapid detection of both Ogawa and Inaba serotypes of O1 | Health posts | 10^7^CFU/mL | 15min |
| **Crystal VC***** | Span Diagnostics Ltd., India | CIA | mAb to *V. cholerae* O1 and O139 LPS | Point of care testing & surveillance | Field & Health posts | 10^7^ CFU/mL | 15-20min |
| **Smart Q** | SmarTest Diagnostics, Israel | CIA | *V. cholerae* O1&O139 | Qualitative detection of O1&O139 in environmental specimens (surfaces or powdered solids) | Health posts | 10^5^ CFU/mL | 20min |
| **Cholera SMART II** | New Horizons Diagnostics Co., USA | CIA | mAb to A antigen of O1 LPS | Direct presumptive detection of *V. cholerae* O1 in stool samples | Health posts in epidemics and endemic areas and peripheral labs in endemic regions | 2x10^7^ CFU/mL | 20min |
| **Cholera O139 SMART II** | New Horizons Diagnostics Co., USA | CIA | mAb to A antigen of O139 LPS | Direct presumptive detection of *V. cholerae* O139 in stool samples | Health posts in epidemics and endemic areas and peripheral labs in endemic regions | 2x10^7^ CFU/mL | 15min |
| **Cholera & Bengal SMART** | New Horizons Diagnostics Co., USA | DFA | *V. cholerae* O1 & O139 | Quantitative for watery & solid stool samples, liquid & solid food samples | Health posts and peripheral labs | 2x10^11^ CFU/mL | 15min |
| **Bengal DFA** | New Horizons Diagnostics Co., USA | DFA | A antigen O139LPS | Direct detection of VC O139 in clinical, food & environmental samples | Peripheral or reference labs | 10^4^CFU/mL | 10min |
| **Cholera O1 DFA** | New Horizons Diagnostics Co., USA | DFA | A antigen O1 LPS | Direct detection of VC O1 in clinical, food & environmental samples | Peripheral or reference labs | 10^4^CFU/mL | <2hr |
| **AccuTest** | DTA Pty Ltd. | Membrane based sandwich CIA | *V. cholerae* | Quantitative for watery & solid stool, liquid & solid food samples | Health posts and peripheral labs | 10^5^CFU/mL | <15min |
| **Cholera Ag O1** | Standard Diagnostics Inc., Korea | CIA | Colloidal-gold-labeled *V. cholerae* O1 antibodies | Detect O1 in humam fecal specimens | Health posts | ** | 10-20min |
| **Cholera Ag O1/O139** | Standard Diagnostics Inc., Korea | CIA | Mouse mAb anti- *V. cholerae* O1 & anti-O139. | Rapid, qualitative detection of O1 and O139 in fecal specimens | Health posts | ** | 10-20min |
| **Cholera spot test** | MBDr, Malaysia. | CIA | ** | *V. cholerae* | Health posts | 10^6^ cfu/ml | <20min |
| **QuickTest Cholera Cassette** | ORGENICS, France | CIA | Cholera O1 Ag | Qualitative determination in human stool samples | Health posts | ** | 10min |

** Information not provided by manufacturer or product information. The following diagnostic tests not discussed above due to absence of information; Rapid Cholera sign (DEVARON), Rapid Cholera Strip (DEVARON), Rapid Cholera Flow (DEVARON), Pathogen detection kit (PDK, Intelligent Monitoring Systems, Gainesville, Florida, USA), RI50/2-00 Cholera Ab Dipstick (A.M.S Diagnostics (previously known as R150/2-00 Megakwik Cholera Ab Card (Discontinued)), BioSign (PBM), Medicos dipstick (Advanced Diagnostics Inc., South Plainfield, NJ, USA), Quix rapid cholera strip test (Guardian Scientific Corp). *** Crystal VC is a more recent manufactured form of the original IP dipstick. The following abbreviations were used: RT-PCR = Real time polymerase chain reaction, *ctx* = cholera enterotoxin, MITD =Medical Innovation & Technology Development INFORMM =Institute for Research in Molecular Medicine USM = Universiti Sains Malaysia, MBDr =Malaysian Biodiagnostics Research Sdn Bhd., RPLA = Reversed passive latex agglutination, COAT = Coagglutination test, LPS = Lipo poly saccharide, CIA = chromatographic Immuno Assay, SMART = Sensitive Membrane Antigen Rapid Test, DFA = Direct Fluorescence Antibody.
