# Supplementary material for: Review of Two Decades of Cholera Diagnostics – How Far Have We Really Come?
Source: PLoS Negl Trop Dis. 2012 Oct 11;6(10):e1845. doi: 10.1371/journal.pntd.0001845 (PMC3469466; doi:10.1371/journal.pntd.0001845)
Supplement: Table S2 — Recent peer-reviewed evaluations of cholera diagnostic tests - Field. (DOC) [file pntd.0001845.s002.doc]

**Table S2. Recent peer-reviewed evaluations of cholera diagnostic tests** - Field

| **Publication** | **Name** | **Study type** | **Population** | | **Diagnostic target** | **Golden standard** | **Sensitivity**  **(Se)** | **Specificity (Sp)** | **PPV** | **NPV** |
| --- | --- | --- | --- | --- | --- | --- | --- | --- | --- | --- |
|  |  |  | **Case** | **Control** |  |  |  |  |  |  |
| **Harris [12]**  Guinea Bissau **2008** | Crystal VC | Field | 101 | ** | O1 and O139 LPS | PCR | 97 | 71-76 | 87-89 | 92-93 |
| **Wang [13]**  Mozambique **2006** | IP O1 cholera dipstick | Field | 391 | ** | O1 LPS | culture | 95 | 89 | 83 | ** |
| **Kalluri [14]**  Bangladesh & USA **2006** | IP dipstick | Field | 304 | ** | O1 LPS | culture | 93 | 67 | 63 | 94 |
| **Kalluri [14]**  Bangladesh & USA **2006** | Medicos | Field | 304 | ** | ** | culture | 84 | 79 | 71 | 90 |
| **Kalluri [14]**  Bangladesh & USA **2006** | SMART | Field | 304 | ** | Antigen A of O1 LPS | culture | 58 | 95 | 84 | 84 |
| **Nato [15]**  Madagascar **2003** | IP cholera dipstick | Field | 140 | 30 | O1 & O139 | culture | 98.5 | 96 | 95.6 | 98.6 |
| **Nato [15]** Bangladesh **2003** | IP cholera dipstick | Field | 102 | 30 | O1LPS | culture | 94.2 | 84 | 86 | 93.3 |
| **Nato [15]** Bangladesh **2003** | IP cholera dipstick | Field | 158 | 30 | O139LPS | culture | 100 | 92.5 | 94.8 | 100 |
| **Hasan [16]**  India & Mexico **1995** | Bengal DFA | laboratory & field | India; 35, Mexico; 93 | 197 | ** | culture | 100 | 100 | 100 | 100 |
| **Hasan [16]**  India & Mexico **1995** | Bengal Screen | laboratory & field | India ; 35, Mexico; 93 | 197 | ** | culture | 95 | 100 | 100 | 0.94 |
| **Islam [17]**  Zaire **1995** | Cholera Screen  & Cholera SMART | Laboratory & field | 30 | ** | O1 | culture | 100 | 100 | N/A | N/A |
| **Arya [18]**  Bangladesh **1995** | COAT | laboratory & field | 120 | ** | ** | culture | 92 | 100 | 100 | 95 |
| **Hasan [19]**  USA & Bangladesh**, 1994** | DFA COLTA | laboratory & field | 44 | 130 | O1 LPS antigen A | culture | 100 | 100 | N/A | N/A |
| **Hasan [20]**  Bangladesh, **1994** | Cholera SMART | field | 44 | 120 | O1 | culture | 95.6 | 100 | ** | ** |
| **Hasan [20]**  Mexico, **1994** | Cholera SMART | field | 108 | 120 | O1 | culture | 100 | 100 | ** | ** |
| **Colwell [21]**  Guatemala, **1992** | Cholera Screen | field | 17 | ** | O1 | culture & DFA | 85.7 | 60 | N/A | N/A |
| **Colwell [21]**  Bangladesh, **1992** | Cholera Screen | field | 77 | ** | O1 | culture | 98 | 77.8 | N/A | N/A |

** Information was not provided. N/A = not applicable, LPS = Lipopolysaccharide, IP = Institute Pasteur, SMART= Sensitive Membrane Antigen Rapid Test, DFA = Direct Fluorescent Antibody, COAT = Coagglutination Tes
