# Supplementary material for: Review of Two Decades of Cholera Diagnostics – How Far Have We Really Come?
Source: PLoS Negl Trop Dis. 2012 Oct 11;6(10):e1845. doi: 10.1371/journal.pntd.0001845 (PMC3469466; doi:10.1371/journal.pntd.0001845)
Supplement: Table S3 — Recent peer-reviewed evaluations of cholera diagnostic tests - Laboratory. (DOC) [file pntd.0001845.s003.doc]

**Table S3. Recent peer-reviewed evaluations of cholera diagnostic tests - Laboratory**

| **Publication** | **Name** | **Study type** | **Population** | | **Diagnostic target** | **Golden standard** | **Sensitivity**  **(Se)** | **Specificity (Sp)** | **PPV** | **NPV** |
| --- | --- | --- | --- | --- | --- | --- | --- | --- | --- | --- |
|  |  |  | **Case** | **Control** |  |  |  |  |  |  |
| **Chan [22]**  Malaysia**, 2008** | Cholera Genosensor | Laboratory | 10 | 21 | lolB (hemM) gene | PCR | 100 | 100 | N/A | N/A |
| **Kalluri [14]**  Bangladesh & USA **2006** | IP dipstick | Laboratory | 304 | ** | O1 LPS | Culture | 94 | 76 | 70 | 95 |
| **Kalluri [14]**  Bangladesh & USA **2006** | Medicos | Laboratory | 304 | ** | ** | culture | 88 | 80 | 72 | 92 |
| **Kalluri [14]**  Bangladesh & USA **2006** | SMART | Laboratory | 304 | ** | Antigen A of O1 LPS | culture | 83 | 88 | 83 | 88 |
| **Nato [15]**  France **2003** | IP cholera dipstick | Laboratory | ** | 30 | O1 LPS | culture | 100 | 100 | N/A | N/A |
| **Nato [15]**  France **2003** | IP cholera dipstick | Laboratory | ** | 30 | O139LPS | culture | 89.5 | 100 | N/A | N/A |
| **Bhuiyan [23]**  Bangladesh **2003** | IP dipstick  O1 | Laboratory (surveillance) | 134 | ** | O1 LPS | culture & multiplex PCR | 96 | 92 | 93 | ** |
| **Bhuiyan [23]**  Bangladesh **2003** | IP dipstick O139 | Laboratory (surveillance) | 134 | ** | O139 LPS | culture & multiplex PCR | 93 | 98 | 93 | ** |
| **Rohani [24]**  Malaysia **1998** | Cholera Spot test | Laboratory | ** | 22 | ** | N/A | 100 | 100 | N/A | N/A |
| **Quadri [25]**  Bangladesh **1995** | Bengal SMART | Laboratory | 189 | ** | ** | COAT & cluture | 100 | 97 | 97 | 100 |
| **Islam [26]**  Bangladesh **1994** | Cholera Screen | Laboratory | 57 | ** | ** | culture | "no false positive reaction” | "highly specific" | too small | too small |
| **Carillo [27]**  Peru**, 1994** | Cholera Screen | laboratory | 100 | 100 | O1 LPS factor A | culture | 99 | 22 | 85 | ** |
| **Carillo [27]**  Peru**, 1994** | latex agglutination | laboratory | 100 | 100 | O1 LPS factor A | culture | 100 | 33 | 87 | ** |
| **Hasan [20]**  USA, **1994** | Cholera SMART | laboratory | ** | 120 | O1 | culture | ** | ** | N/A | N/A |
| **Colwell [21]**  USA, **1992** | Cholera Screen | laboratory | 30 | 88 | O1 | culture | 100 | 100 | N/A | N/A |
| **Yam [28]**  Hong Kong**, 1992** | VET-RPLA | laboratory | 56 | 18 | CT | ** | 75 | 100 | N/A | N/A |
| **Almeida [29]**  Japan **1990** | VET-RPLA | laboratory | 88 | 80 | CT | ELISA | 97 | 100 | N/A | N/A |

** Information was not provided. N/A = not applicable, LPS = Lipopolysaccharide, IP = Institute Pasteur, SMART= Sensitive Membrane Antigen Rapid Test, VET-RPLA = Reverse-Passive Latex Agglutination, COAT = Coagglutination Test, CT=Cholera Toxin
